# How Does Exercise, With and Without Diet, Improve Pain and Function in Knee Osteoarthritis? A Secondary Analysis of a Randomized Controlled Trial Exploring Potential Mediators of Effects

**DOI:** 10.1002/acr.25140

**Published:** 2023-06-15

**Authors:** Belinda J. Lawford, Rana S. Hinman, Fiona McManus, Karen E. Lamb, Thorlene Egerton, Catherine Keating, Courtney Brown, Kathryn Oliver, Kim L. Bennell

**Affiliations:** ^1^ The University of Melbourne Melbourne Victoria Australia; ^2^ Medibank Private Melbourne Victoria Australia

## Abstract

**Objective:**

To explore the mediators of effects of two 6‐month telehealth‐delivered exercise programs, including exercise with and without weight‐loss diet, on pain and function improvements in knee osteoarthritis (OA).

**Methods:**

Secondary analysis of 345 participants from a 3‐arm randomized controlled trial of exercise (Exercise program) and exercise plus diet (Diet + Exercise program) versus information (Control program) was conducted. Outcomes were changes in pain (11‐point numeric rating scale) and function (Western Ontario and McMaster Universities Osteoarthritis Index [score range 0–68]) at 12 months. Potential mediators were change at 6 months in attitudes toward self‐management, fear of movement, arthritis self‐efficacy, weight, physical activity, and willingness for knee surgery. For the Diet + Exercise program versus the Exercise program, only change in weight was evaluated.

**Results:**

Possible mediators of the Exercise program versus the Control program included reduced fear of movement (accounting for –1.11 units [95% confidence interval (95% CI) –2.15, –0.07] improvement in function) and increased arthritis self‐efficacy (–0.40 units [95% CI –0.75, –0.06] reduction in pain, –1.66 units [95% CI –3.04, –0.28] improvement in function). The Diet + Exercise program versus the Control program mediators included reduced fear of movement (–1.13 units [95% CI –2.17, –0.08] improvement in function), increased arthritis self‐efficacy (–0.77 units [95% CI –1.26, –0.28] reduction in pain, –5.15 units [95% CI –7.34, –2.96] improvement in function), and weight loss (–1.20 units [95% CI –1.73, –0.68] reduction in pain, –5.79 units [95% CI –7.96, –3.63] improvement in function). Weight loss mediated the Diet + Exercise program versus the Exercise program (–0.89 units [95% CI –1.31, –0.47] reduction in pain, –4.02 units [95% CI –5.77, –2.26] improvement in function).

**Conclusion:**

Increased arthritis self‐efficacy, reduced fear of movement, and weight loss may partially mediate telehealth‐delivered exercise program effects, with and without diet, on pain and/or function in knee OA. Weight loss may partially mediate the effect of diet and exercise compared to exercise alone.

## Graphical abstract

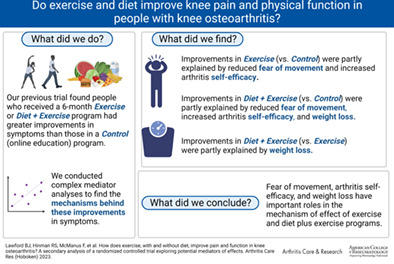

## INTRODUCTION

All current clinical guidelines recommend education, exercise, and weight loss (if indicated) as first‐line management approaches for knee osteoarthritis (OA) (1, 2, 3, 4). In those individuals who are overweight or obese, there is evidence that combining a weight‐loss diet with exercise is optimal, with benefits of the combination exceeding the effects of either treatment alone (5, 6). A recent systematic review and meta‐analysis found that diet‐induced weight loss alone did not improve pain for people with knee OA who were overweight or obese, but there were moderate effects from interventions that combined diet and exercise (7). As such, scalable interventions that combine weight‐loss diet with exercise may help maximize clinical outcomes and reduce the enormous individual and societal burden of the condition (8).

**SIGNIFICANCE & INNOVATIONS**

- Our previous three‐arm randomized controlled trial found that two 6‐month telehealth‐delivered programs, including exercise with and without weight‐loss diet, led to improvements in pain and physical function in people with knee osteoarthritis compared to information‐only control. In this study, we used causal mediation analyses to explore potential mediators of these effects.
- Reduced fear of movement and increased arthritis self‐efficacy may mediate the effects of exercise, compared to control, on pain and/or function.
- Reduced fear of movement, increased arthritis self‐efficacy, and weight loss may mediate the effects of exercise with weight‐loss diet, compared to control, on pain and/or function.
- Weight loss may mediate the effects of exercise with weight‐loss diet, compared to exercise alone, on pain and function.

Despite clinical guidelines recommending diet and exercise for management of knee OA, the clinical benefits of these treatments for this population are only modest (1, 2, 3, 4, 7). This may be partly due to limited understanding about the mechanisms by which exercise and diet approaches work to improve pain and physical function. Identifying precise mechanisms of effect will help ensure future treatment programs are designed to target these mechanisms, potentially leading to enhanced effects on clinical outcomes such as pain and function. A robust method of identifying mechanisms of effect is through causal mediation analysis using data from a randomized controlled trial (RCT). Causal mediation analyses examine the causal links between an intermediate variable (mediator) and the effect of an intervention on outcomes (9). A recent scoping review of mediation analysis studies examining nonsurgical interventions for people with OA found that reduced inflammation, reduced body weight, increased muscle strength, and increased self‐efficacy may mediate effects of nonsurgical interventions on pain and physical function (10). Specifically, increased knee extensor muscle strength (11) and knee flexor muscle perfusion (12) partially mediated the effects of exercise on changes in pain and physical function in adults with knee OA, but no previous studies had examined psychosocial putative mediators of exercise like self‐efficacy or fear of movement. In combined diet and exercise programs, there was inconsistent evidence that weight loss (13), inflammatory biomarkers (14), self‐efficacy (for walking duration [15] and for OA symptoms [16]), and pain control (perceived ability to exert control over one's pain) (16) mediate the effects of the programs on pain and physical function. Given the paucity and heterogeneity of existing evidence identified by authors of the review (10), more research is required to identify potential mediators of exercise interventions, including those with and without diet, for people with OA.

Recently, our RCT found that two 6‐month telehealth‐delivered programs, including exercise with and without weight‐loss diet, led to improvements in pain and physical function at 6 months and 12 months, compared to a control group who received online information (6, 17). Compared to the exercise‐only group, the combined diet and exercise program led to modest additional improvements in pain and function. However, from the RCT alone, the mechanisms underpinning the effectiveness of both the diet and exercise and exercise‐only programs on symptoms are not clear. Using data from our RCT (6), we used causal mediation analysis to explore potential mediators of the effects of our 2 exercise programs, one with and one without weight‐loss diet, on improvements in pain and physical function in people with knee OA who are overweight/obese.

## MATERIALS AND METHODS

This is a secondary analysis of data from an RCT comparing the effects of exercise, with and without a weight‐loss diet, to an information‐only control group in people with knee OA (Australian New Zealand Clinical Trials Registry ACTRN12618000930280) (6, 17). All participants provided written informed consent and The University of Melbourne Human Research Ethics Committee approved the study.

### Participants

Participants were recruited from members of Australia's largest private health insurer, Medibank Private. Medibank sent targeted invitations, predominantly via email, to members. Eligible participants including the those who: 1) held private health insurance with Medibank at a level that included cover for arthroplasty surgery; 2) met the National Institute for Health and Care Excellence OA clinical criteria (ages ≥45 years, activity‐related joint pain, morning stiffness ≤30 minutes) (3); 3) had average knee pain ≥4 on 11‐point numerical rating scale (NRS) in the past week (0 = no pain, 10 = worst pain possible); 4) had a history of knee pain on most days for at least 3 months; 5) were ages <81 years; and 6) had a body mass index ≥28 kg/m^2^ and <41 kg/m^2^. Detailed inclusion and exclusion criteria for the RCT are published (17).

### Control group

Participants in the Control group were given access to a bespoke website that contained information about OA, treatment options, exercise and physical activity, weight loss, managing pain, sleep, and “success stories.” The website also provided links to external websites for further information.

### Interventions

The intervention protocol was previously published (17). All project clinicians underwent training prior to the start of the trial, including in best‐practice management of OA, motivational interviewing skills, specifics of the weight‐loss diet, and study‐specific protocols (17).

### Exercise program

Participants in the Exercise group had 6 individual videoconferencing consultations (Zoom Video Communications Inc.) with a physical therapist over 6 months where they were prescribed a strengthening exercise and physical activity program. Initial consultations lasted ~45 minutes, with follow‐up consultations lasting ~20 minutes. Physical therapists also provided individualized advice about treatment options and used motivational interviewing principles to support behavior change. Participants received hard copy information booklets (information about OA, exercise instructions, log book), exercise bands, and a Fitbit (Flex 2 model) to monitor physical activity.

### Diet + Exercise program

Participants in the Diet + Exercise group received all components of the Exercise group, plus 6 individual videoconferencing consultations with a dietitian over 6 months to guide them through a ketogenic very low‐calorie diet (VLCD). This diet involved consuming ~800 calories (or 3,280 kilojoules) per day (18) and replacing 2 meals per day with Optifast meal replacements (Nestlé Health Science) (or Optislim [OptiPharm Pty Ltd] if unavailable or if the participant was vegetarian). Participants were encouraged to lose at least 10% of their body weight on the diet (5) before transitioning off meal replacements to a healthy eating diet. Initial consultations lasted ~45 minutes, with follow‐up consultations lasting ~20 minutes. Dietitians used motivational interviewing to help participants adhere to their weight management plan. Participants received additional weight management booklets (“how to” guide, recipe book, weight management activities), a plastic portion plate, and up to a 6‐month supply of meal replacements.

### Outcomes

Outcomes were self‐reported via online questionnaires at baseline, 6 months, and 12 months. Outcomes relevant to this mediation analysis include the primary outcomes of change in knee pain and physical function at 12 months. Overall knee pain was measured using an 11‐point NRS ranging from 0 (no pain) to 10 (worst pain possible). Physical function was measured using the Western Ontario and McMaster Universities Osteoarthritis Index (WOMAC OA Index) function subscale (19), with scores ranging from 0 (no dysfunction) to 68 (maximum dysfunction).

### Mediator variables

Six potential mediator variables were measured at baseline and 6 months:

1. Attitudes towards self‐management, measured using the Patient Activation Measure (20) (score range 13–42, with higher scores indicating greater patient activation);
2. Fear of movement, measured using the Brief Fear of Movement Scale for Osteoarthritis (21) (score range 6–24, with higher scores indicating greater fear);
3. Self‐efficacy for managing arthritis symptoms, measured using the Arthritis Self‐Efficacy Scale (22) (score range 3–30, with higher scores indicating greater self‐efficacy);
4. Body weight in kilograms, which was self‐reported;
5. Physical activity, measured using the Incidental and Planned Exercise Questionnaire, “past week” version (23) (score range 0–128, with higher scores indicating higher levels of activity); and
6. Willingness to have surgery, rated on a 5‐point scale ranging from “definitely not willing” to “definitely willing,” with those indicating “probably not willing” or “definitely not willing” classified as unwilling to have knee surgery in the near future and all other options classified as willing.

### Statistical analysis

All statistical analyses were performed using Stata, version 16.1 (StataCorp LLC), on complete case data (i.e., excluding participants with any missing baseline data, missing outcome data [knee pain or physical function] at 12 months, and missing potential mediator data at 6 months). Complete case data were used in this exploratory study as characteristics of the complete case and omitted sample were comparable (see Supplementary Table 1, available on the *Arthritis Care & Research* website at http://onlinelibrary.wiley.com/doi/10.1002/acr.25140). Regression assumptions of linearity and homoscedasticity were assessed using standard diagnostic plots.

**Figure 1:**
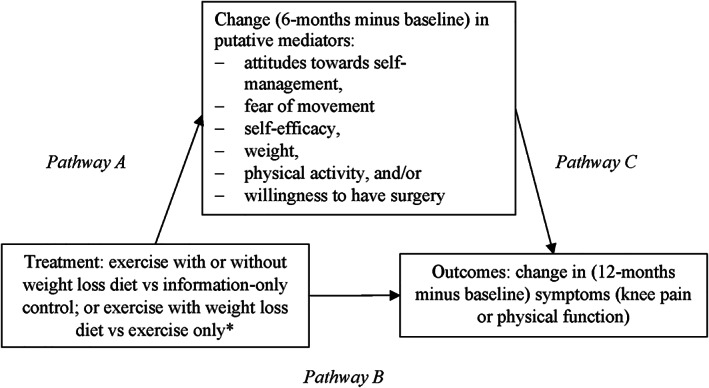
The effect of treatment on potential mediators (Pathway A), the direct effect of treatment on outcomes (Pathway B), and the effect of potential mediators on outcomes (Pathway C). Total effect is the sum of direct effect (Pathway B) and indirect effect (Pathway A multiplied by Pathway C). * For the Diet + Exercise versus Exercise comparison, only change (6 months minus baseline) in weight was considered.

Initially, to explore the effect of the treatment group on each continuous and binary potential mediator, respectively, at 6 months, separate linear regression and logistic regression models were fitted (Figure 1, Pathway A). This was conducted separately for each pair of treatment groups, Diet + Exercise versus Control and Exercise versus Control. For Diet + Exercise versus Exercise, a linear regression model was fitted only on the potential mediator, change in weight. Results were calculated as the estimated mean (95% confidence interval [95% CI]) difference in change (6 months minus baseline) in each continuous potential mediator between groups. The estimated relative risk and risk difference (95% CI) in the binary mediator (unwillingness to have surgery at 6 months) between groups were calculated.

Next, to explore if mediation was present, full causal mediation analyses (based on the potential outcomes framework and the counterfactual framework [24, 25]) were conducted where 2 regression models were simultaneously fitted for each outcome (change in knee pain and change in physical function), considering each potential mediator and each relevant treatment group comparison separately. Potential mediators were investigated separately as this was an exploratory study, and there is a paucity of evidence regarding psychosocial mediators of exercise and diet. The first of the 2 regression models estimated the direct effect of the first named group in the pairwise comparison (e.g., Diet + Exercise in Diet + Exercise versus Control comparison) and the effect of the potential mediator on the outcome (Figure 1, Pathways B and C). The second model estimated the effect of the first named group in the pairwise comparison on the potential mediator (Figure 1, Pathway A). These 2 models permitted the total effect of the first named group in the pairwise comparison on the outcome to be decomposed into the direct effect (Figure 1, Pathway B) and the indirect effect (Figure 1, Pathway A multiplied by C). The direct effect refers to the effect of the first named group in the pairwise comparison on the outcome that does not occur through the potential mediator. The indirect effect is the effect of the first named group that does occur through (i.e., is mediated by) the potential mediator.

Estimating direct and indirect effects using causal mediation analysis assumes that: 1) there are no unmeasured treatment–outcome confounders; 2) there are no unmeasured mediator–outcome confounders; 3) there are no unmeasured treatment–mediator confounders; and 4) there is no effect of treatment that confounds the mediator–outcome relationship (26, 27). As treatment was randomly allocated and it appears reasonable that missing data were missing completely at random, assumptions 1 and 3 are satisfied. History of knee surgery (the stratification variable in the original trial), baseline mediator and baseline outcome scores (for the specific mediator and outcome considered in that model) were included as covariates in both regression models, as these were assumed to be the only potential confounders of the potential mediator–outcome relationship (Figure 1, Pathway C). There were no effects of treatment known to the authors a priori that could confound each mediator–outcome relationship (assumption 4). These causal mediation analyses were each conducted using the “paramed” function (28). The “medeff” function in Stata (29) was used to calculate the proportion of the total effect mediated through the potential mediator, estimated as the ratio of the indirect effect to the total effect, and presented as a percentage (95% CI). Percentages can exceed 100% if the sum of indirect effects exceeds total effects, which occurs if mediators affect one another or if there are interactions between mediators (30).

Finally, to estimate the magnitude of the association between each potential mediator (6 months minus baseline or at 6 months) and change in each symptom (12 months minus baseline), separate linear regression models were used (Figure 1, Pathway C). In these models, change in symptoms was entered as the outcome, the potential mediator was the independent variable and relevant baseline mediator scores, relevant baseline outcome scores, the stratifying variable, and each relevant pair of treatment groups were entered as covariates. Results were calculated as the estimated mean (95% CI) effect of the potential mediator on change in symptoms (12 months minus baseline). Analyses for Pathways A and C in Figure 1 are provided in Supplementary Table 6, (available on the *Arthritis Care & Research* website at http://onlinelibrary.wiley.com/doi/10.1002/acr.25140). However, these do not influence the causal mediation analyses. Therefore, the results are not described further (30).

We did not adjust for multiplicity as this was an exploratory study, where all results are hypothesis‐generating and not confirmatory. We have reported all effects, confidence intervals, and *P* values to let readers use their own judgment about the relative weight of the conclusions. This approach aligns with the usage of *P* values favored by the American Statistical Association (31).

Sensitivity analyses (Supplementary Table 2, available at http://onlinelibrary.wiley.com/doi/10.1002/acr.25140) for mediation effects were conducted to investigate how robust the full causal mediation analyses results were to violation of the sequential ignorability assumption (i.e., that there is an unmeasured confounder related to both the mediator and the outcome) (32). Violation was assessed using a sensitivity parameter, rho, which represents the correlation between the error terms of the mediator and outcome models, a measure of the degree of unmeasured mediator–outcome confounding (29). This parameter was allowed to vary to determine the impact on the resulting estimated indirect (mediation) effect, then the value of rho at which this effect is zero was examined.

## RESULTS

Of the 415 participants enrolled in the trial, 345 (83%) had complete case data and were analyzed in this mediation analysis. The control group had a higher proportion of female participants, otherwise baseline characteristics of participants were similar between groups (Table 1).

**Table 1:** Participant baseline characteristics*

	Control	Exercise	Diet + Exercise	Missing data
	(n = 45)	(n = 137)	(n = 163)	(n = 69)†
Age, mean ± SD years	64.9 ±8.7	66.0 ± 8.2	64.2 ± 8.2	64.0 ± 8.1
Female, no. (%)	31 (68.9)	77 (56.2)	85 (52.1)	34 (49.3)
Body mass index (kg/m^2^)	33.2 (30.8–36.5)	32.0 (29.9–34.6)	32.4 (30.4–35.7)	34.6 (31.6–37.6)
Knee pain (NRS)	6 (5–7)	6 (5–7)	6 (5–7)	6 (5–6)
Physical function (WOMAC), mean ± SD	21.2 ± 9.9	22.2 ± 10.7	24.1 ± 9.0	24.7 ± 10.0
Attitudes towards self‐management (PAM‐13)	46 (40–49)	44 (40–49)	44 (40–48)	44 (40–47)
Fear of movement (BFMS)	11 (10–14)	12 (9–14)	12 (10–15)	13 (10–14)
Self‐efficacy (ASES), mean ± SD	21.4 ± 3.5	20.7 ± 4.1	20.6 ± 3.7	19.4 ± 3.7
Weight (kg), mean ± SD	96.6 ± 13.6	94.1 ± 13.0	95.4 ± 13.6	99.5 ± 15.3
Physical activity (IPEQ)	17.1 (11.9–35.8)	22.8 (12.9–34.0)	21.6 (11.9–32.3)	16.6 (11.0–29.4)
Unwilling to have surgery, no. (%)‡	12 (26.7)	42 (30.7)	45 (27.6)	16 (23.2)
History of knee surgery (arthroscopy or contralateral arthroplasty), no. (%)	26 (57.8)	80 (58.4)	93 (57.1)	35 (50.7)

* Values are the median (interquartile range [IQR]) unless indicated otherwise. ASES = Arthritis Self‐Efficacy Scale (scored 3–30, with higher scores indicating greater self‐efficacy); BFMS = Brief Fear of Movement Scale for osteoarthritis (scored 6–24, with higher scores indicating greater fear); IPEQ = Incidental and Planned Exercise Questionnaire (version W; scored 0–128, with higher scores indicating higher levels of activity); NRS = numerical rating scale (rated 0–10, with higher scores indicating worse pain); PAM‐13 = Patient Activation Measure (scored 13–52, with higher scores indicating greater patient activation); WOMAC = Western Ontario and McMaster Universities Osteoarthritis Index (physical function subscale; rated 0–68, with higher scores indicating worse function).

† One participant requested withdrawal of all data from the study (i.e., of 415 participants enrolled, data for 414 participants are shown).

‡ Rated using a 5‐point scale with terminal descriptors of “definitely not willing” to “definitely willing,” with those indicating “probably not willing” or “definitely not willing” classified as unwilling to have knee surgery in the near future and all other options classified as willing.

### Effect of treatment group on potential mediators

Results for Pathway A, the effect of treatment on potential mediators, are shown in Tables 2 and 3. Compared to the Control program, the Exercise program treatment led to improvements in fear of movement, self‐efficacy, reduction in weight, physical activity, and increased unwillingness to have surgery. Compared to the Control program, the Diet + Exercise program led to improvements in attitudes toward self‐management, fear of movement, self‐efficacy, reduction in weight, and increased unwillingness to have surgery. The Diet + Exercise program led to a reduction in weight compared to the Exercise program.

**Table 2:** Mean ± SD change by group and mean (95% CI) difference in change for each potential mediator between groups*

Potential mediators	Control (n = 45)†	Exercise (n = 137)†	Diet + Exercise (n = 163)†	Exercise vs. Control (n = 182)‡	*P*	Diet + Exercise vs. Control (n = 208)‡	*P*	Diet + Exercise vs. Exercise (n = 300)‡	*P*
Attitudes toward self‐management (PAM‐13)¶	0.16 ± 6.53	1.44 ± 6.62	3.25 ± 6.29	1.33 (–0.50, 3.17)	0.154	2.88 (1.00, 4.75)	0.003	–	–
Fear of movement (BFMS)§	–0.13 ± 3.42	–1.67 ± 3.30	–2.06 ± 4.04	–1.61 (–2.56, –0.66)	<0.001	–1.61 (–2.73, –0.49)	0.005	–	–
Self‐efficacy (ASES)¶	–0.51 ± 3.59	2.95 ± 5.73	4.81 ± 4.66	3.01 (1.43, 4.59)	<0.001	4.77 (3.51, 6.03)	<0.001	–	–
Weight (kg)§	–0.52 ± 4.12	–1.71 ± 3.72	–10.29 ± 6.05	–1.36 (–2.62, –0.09)	0.035	–9.95 (–11.69, –8.21)	<0.001	–8.39 (–9.49, –7.30)	<0.001
Physical activity (IPEQ‐W)¶	0.43 ± 13.45	6.49 ± 18.62	5.25 ± 16.71	5.87 (0.54, 11.19)	0.031	4.40 (–0.36, 9.16)	0.070	–	–

* Mean (95% CI) difference in change (6 months minus baseline) in each potential mediator between treatment groups, adjusted for baseline mediator scores and the stratifying variable, history of knee surgery (arthroscopy or contralateral arthroplasty), estimated using separate regression models for each treatment group comparison. 95% CI = 95% confidence interval; ASES = Arthritis Self‐Efficacy Scale (scored 3–30, with higher scores indicating greater self‐efficacy); BFMS = Brief Fear of Movement Scale for osteoarthritis (scored 6–24, with higher scores indicating greater fear); IPEQ‐W = Incidental and Planned Exercise Questionnaire (“past week” version; scored 0–128, with higher scores indicating higher levels of activity); PAM‐13 = Patient Activation Measure (scored 13–52, with higher scores indicating greater patient activation).

† Values are the mean ± SD change within groups (6 months minus baseline) (positive changes indicate improvement).

‡ Values are the mean (95% confidence interval [95% CI]) difference in change (6 months minus baseline) between groups (positive differences favor the first named group in the pairwise comparison).

§ For change within groups, negative changes indicate improvement. For difference in change between groups, negative differences favor the first named group in the pairwise comparison.

¶ For change within groups, positive changes indicate improvement. For difference in change between groups, positive differences favor the first named group in the pairwise comparison.

**Table 3:** Counts (proportions) at 6 months by group and relative risks and risk differences for each potential mediator between groups*

Counts (proportions) at 6 months	Control (n = 45	Exercise (n = 137)	Diet + Exercise (n = 163)	Exercise vs. Control (n = 182)	*P*	Diet + Exercise vs. Control (n = 208)	*P*	Diet + Exercise vs. Exercise (n = 300)	*P*
Unwilling to have surgery, values†									
Mean ± SD	19 ± 42.2	84 ± 61.3	116 ± 71.2	–	–	–	–	–	–
Relative risk (95% CI)‡	–	–	–	1.40 (1.00, 1.97)	0.047	1.66 (1.22, 2.26)	0.001	–	–
Risk difference (95% CI)§	–	–	–	0.18 (0.02, 0.33)	0.027	0.28 (0.14, 0.42)	<0.001	–	–

* Relative risks and risk differences (95% confidence interval [95% CI]) estimated using separate logistic regression models for each treatment group comparison, adjusted for baseline mediator scores and the stratifying variable, history of knee surgery (arthroscopy or contralateral arthroplasty).

† Rated using a 5‐point scale with terminal descriptors of “definitely not willing” to “definitely willing,” with those indicating “probably not willing” or “definitely not willing” classified as unwilling to have knee surgery in the near future, and all other options classified as willing.

‡ Relative risks >1 favor the first named group in the pairwise comparison.

§ Risk differences >0 favor the first named group in the pairwise comparison.

### Mediators of effects of Exercise compared to Control

The full causal mediation analysis for the effects of the Exercise program, compared to the Control program, on outcomes is shown in Table 4 and Supplementary Table 7 (available on the *Arthritis Care & Research* website at http://onlinelibrary.wiley.com/doi/10.1002/acr.25140). For change in knee pain, the effect of the Exercise program versus the Control program was only mediated by change in self‐efficacy. The total effect of the Exercise program versus the Control program was a 0.70‐unit reduction on the NRS (95% CI –1.45, 0.05). An estimated 0.40 units (–0.75, –0.06) of that reduction was through an increase in self‐efficacy (corresponding to 54% of the total effect).

**Table 4:** Estimated mean (95% CI) total, direct, and indirect effects of the Exercise program on change in symptoms (12 months minus baseline) compared to the Control program (n = 182)*

Potential mediator†	Total effect, mean (95% CI)	*P*	Direct effect, mean (95% CI)	*P*	Indirect effect, mean (95% CI)	*P*	% mediated (95% CI)‡
Knee pain (NRS)							
Attitudes towards self‐management (PAM‐13)	–0.69 (–1.44, 0.05)	0.067	–0.61 (–1.35, 0.13)	0.108	–0.09 (–0.28, 0.10)	0.367	12 (–52, 79)
Fear of movement (BFMS)	–0.68 (–1.43, 0.06)	0.070	–0.49 (–1.27, 0.29)	0.218	–0.20 (–0.42, 0.03)	0.089	28 (–94, 171)
Self‐efficacy (ASES)	–0.70 (–1.45, 0.05)	0.068	–0.29 (–1.04, 0.46)	0.443	–0.40 (–0.75, –0.06)	0.023	54 (–183, 496)
Weight (kg)	–0.68 (–1.42, 0.05)	0.069	–0.58 (–1.31, 0.16)	0.124	–0.11 (–0.28, 0.07)	0.228	15 (–86, 96)
Physical activity (IPEQ‐W)	–0.70 (–1.45, 0.05)	0.069	–0.61 (–1.37, 0.15)	0.116	–0.09 (–0.25, 0.07)	0.268	11 (–45, 77)
Unwilling to have surgery§	–0.70 (–1.45, 0.05)	0.066	–0.64 (–1.38, 0.10)	0.091	–0.06 (–0.23, 0.10)	0.461	6 (–22, 39)
Physical function (WOMAC)							
Attitudes toward self‐management (PAM‐13)	–4.88 (–8.01, –1.75)	0.002	–4.71 (–7.81, –1.62)	0.003	–0.17 (–0.80, 0.47)	0.603	3 (2, 9)
Fear of movement (BFMS)	–4.79 (–7.92, –1.65)	0.003	–3.68 (–6.78, –0.58)	0.020	–1.11 (–2.15, –0.07)	0.037	23 (14, 60)
Self–efficacy (ASES)	–4.99 (–8.13, –1.85)	0.002	–3.33 (–6.42, –0.23)	0.035	–1.66 (–3.04, –0.28)	0.018	33 (20, 92)
Weight (kg)	–4.68 (–7.85, –1.51)	0.004	–4.37 (–7.48, –1.26)	0.006	–0.31 (–1.02, 0.40)	0.392	7 (4, 19)
Physical activity (IPEQ‐W)	–4.90 (–8.08, –1.72)	0.003	–4.66 (–7.78, –1.55)	0.003	–0.24 (–0.80, 0.32)	0.407	5 (3, 13)
Unwilling to have surgery§	–4.96 (–8.16, –1.76)	0.002	–4.85 (–7.98, –1.73)	0.002	–0.11 (–0.73, 0.52)	0.738	1 (1, 3)

* Adjusted for baseline mediator scores, baseline outcome scores, and the stratifying variable, history of knee surgery (arthroscopy or contralateral arthroplasty); negative effects favor the Exercise group. 95% CI = 95% confidence interval; ASES = Arthritis Self‐Efficacy Scale (scored 3–30, with higher scores indicating greater self‐efficacy); BFMS = Brief Fear of Movement Scale for osteoarthritis (scored 6–24, with higher scores indicating greater fear); IPEQ‐W = Incidental and Planned Exercise Questionnaire (“past week” version; scored 0–128, with higher scores indicating higher levels of activity); NRS = numerical rating scale (rated 0–10, with higher scores indicating worse pain); PAM‐13 = Patient Activation Measure (scored 13–52, with higher scores indicating greater patient activation); WOMAC = Western Ontario and McMaster Universities Osteoarthritis Index (physical function subscale; rated 0–68, with higher scores indicating worse function).

† Potential mediator is change in (6 months minus baseline) except for the binary mediator, unwilling to have surgery, which is at 6 months.

‡ If the sum of the proportion mediated exceeds 100%, then it is likely the mediators affect one another, or there are interactions between the mediators (30).

§ Rated using a 5‐point scale with terminal descriptors of “definitely not willing” to “definitely willing,” with those indicating “probably not willing” or “definitely not willing” classified as unwilling to have knee surgery in the near future, and all other options classified as willing.

For change in physical function, the effect of the Exercise program versus the Control program was mediated by changes in both fear of movement and self‐efficacy. The total effect of the Exercise program versus the Control program was a 4.99‐unit reduction on the WOMAC (–8.13, –1.85). An estimated 1.11 units (–2.15, –0.07) of that reduction was through a reduction in fear of movement, (corresponding to 23% of the total effect), and 1.66 units (–3.04, –0.28) was through an increase in self‐efficacy (corresponding to 33% of the total effect).

### Mediators of effects of Diet + Exercise compared to Control

The full causal mediation analysis for effects of the Diet + Exercise program compared to the Control program on outcomes is shown in Table 5 and Supplementary Table 7 (available on the Arthritis Care & Research website at http://onlinelibrary.wiley.com/doi/10.1002/acr.25140). The effect of the Diet + Exercise program versus the Control program on knee pain was mediated by an increase in self‐efficacy and reduction in weight. The total effect of the Diet + Exercise program versus the Control program was a 1.25‐point reduction on the NRS (–1.95, –0.54). An estimated 0.77 units (–1.26, –0.28) of that reduction was through an increase in self‐efficacy (63% of the total effect) and 1.20 units (–1.73, –0.68) was through a reduction in weight (96% of the total effect).

**Table 5:** Estimated mean (95% CI) total, direct, and indirect effects of the Diet + Exercise program on change in symptoms (12 months minus baseline) compared to Control program (n = 208)*

Potential mediator†	Total effect, mean (95% CI)	*P*	Direct effect, mean (95% CI)	*P*	Indirect effect, mean (95% CI)	*P*	% mediated (95% CI)‡
Knee pain (NRS)							
Attitudes towards self‐management (PAM‐13)	–1.29 (–2.01, –0.57)	<0.001	–1.19 (–1.90, –0.48)	0.001	–0.10 (–0.31, 0.11)	0.365	8 (5, 15)
Fear of movement (BFMS)	–1.31 (–2.02, –0.61)	<0.001	–1.23 (–1.95, –0.51)	<0.001	–0.08 (–0.24, 0.08)	0.301	7 (4, 13)
Self‐efficacy (ASES)	–1.25 (–1.95, –0.54)	<0.001	–0.48 (–1.25, 0.29)	0.226	–0.77 (–1.26, –0.28)	0.002	63 (40, 123)
Weight (kg)	–1.27 (–1.97, –0.57)	<0.001	–0.06 (–0.89, 0.76)	0.878	–1.20 (–1.73, –0.68)	<0.001	96 (63, 190)
Physical activity (IPEQ‐W)	–1.29 (–2.00, –0.57)	<0.001	–1.23 (–1.93, –0.53)	<0.001	–0.05 (–0.15, 0.04)	0.269	4 (3, 8)
Unwilling to have surgery§	–1.31 (–2.03, –0.60)	<0.001	–1.07 (–1.76, –0.38)	0.002	–0.24 (–0.51, 0.02)	0.070	13 (8, 27)
Physical function (WOMAC)							
Attitudes towards self‐management (PAM‐13)	–7.57 (–10.44, –4.70)	<0.001	–6.81 (–9.63, –3.99)	<0.001	–0.76 (–1.97, 0.45)	0.216	10 (7, 16)
Fear of movement (BFMS)	–7.61 (–10.44, –4.78)	<0.001	–6.48 (–9.37, –3.60)	<0.001	–1.13 (–2.17, –0.08)	0.035	14 (10, 21)
Self‐efficacy (ASES)	–7.54 (–10.42, –4.67)	<0.001	–2.39 (–5.35, 0.56)	0.113	–5.15 (–7.34, –2.96)	<0.001	69 (49, 109)
Weight (kg)	–7.41 (–10.19, –4.62)	<0.001	–1.61 (–4.96, 1.74)	0.345	–5.79 (–7.96, –3.63)	<0.001	79 (57, 125)
Physical activity (IPEQ‐W)	–7.58 (–10.49, –4.67)	<0.001	–7.38 (–10.28, –4.47)	<0.001	–0.20 (–0.65, 0.25)	0.378	3 (2, 4)
Unwilling to have surgery§	–7.67 (–10.66, –4.68)	<0.001	–6.55 (–9.46, –3.64)	<0.001	–1.12 (–2.30, 0.05)	0.061	10 (7, 16)

* Estimated mean adjusted for baseline mediator scores, baseline outcome scores and the stratifying variable, history of knee surgery (arthroscopy or contralateral arthroplasty); for total, direct, and indirect effects. Negative effects favor the Diet + Exercise group. ASES = Arthritis Self‐Efficacy Scale; scored 3–30, with higher scores indicating greater self‐efficacy. BFMS = Brief Fear of Movement Scale for osteoarthritis (scored 6–24, with higher scores indicating greater fear); IPEQ‐W = Incidental and Planned Exercise Questionnaire, “past week” version (scored 0–128, with higher scores indicating higher levels of activity); NRS = numerical rating scale; rated 0–10, with higher scores indicating worse pain; PAM‐13 = Patient Activation Measure (scored 13–52, with higher scores indicating greater patient activation); WOMAC = Western Ontario and McMaster Universities Osteoarthritis Index (physical function subscale; rated 0–68, with higher scores indicating worse function).

† Potential mediator is change in (6 months minus baseline) except for the binary mediator, unwilling to have surgery, which is at 6 months.

‡ If the sum of the proportion mediated exceeds 100%, then one of the following must be true: 1) There are other mediators with a negative proportion mediated, 2) the mediators affect one another, or 3) there are interactions between the mediators (30).

§ Rated using a 5‐point scale with terminal descriptors of “definitely not willing” to “definitely willing,” with those indicating “probably not willing” or “definitely not willing” classified as unwilling to have knee surgery in the near future, and all other options classified as willing.

For physical function, the effect of the Diet + Exercise program versus the Control program was mediated by change in fear of movement, self‐efficacy, and weight. The total effect of the Diet + Exercise program versus the Control program was a 7.54‐point reduction on the WOMAC (–10.42, –4.67). An estimated 1.13 units (–2.17, –0.08) of that reduction was through a reduction in fear of movement (14% of the total effect), 5.15 units (–7.34, –2.96) was through an increase in self‐efficacy (69% of the total effect), and 5.79 units (–7.96, –3.63) was through a reduction in weight (79% of the total effect).

### Mediators of effects of Diet + Exercise compared to Exercise

The full causal mediation analysis for effects of the Diet + Exercise program on outcomes, compared to the Exercise program, is shown in Table 6. For knee pain and physical function, the effects of the Diet + Exercise program versus the Exercise program were both mediated by change in weight. The total effect of the Diet + Exercise program versus the Exercise program was a 0.56‐point reduction in pain on the NRS (–1.05, –0.07), of which an estimated 0.89 units (–1.31, –0.47) was through a change in weight. The total effect of the Diet + Exercise program versus the Exercise program was a 3.09‐point reduction in physical dysfunction on the WOMAC (–5.18, –0.99), of which an estimated 4.02 units (–5.77, –2.26) was through a change in weight.

**Table 6:** Estimated mean (95% CI) total, direct, and indirect effects of the Diet + Exercise program on change in symptoms (12 months minus baseline) compared to Exercise program (n = 300)*

Potential mediator†	Total effect, mean (95% CI)	*P*	Direct effect, mean (95% CI)	*P*	Indirect effect, mean (95% CI)	*P*	% mediated, (95% CI)‡
Knee pain (NRS)							
Weight (kg)	–0.56 (–1.05, –0.07)	0.024	0.33 (–0.32, 0.99)	0.317	–0.89 (–1.31, –0.47)	<0.001	–
Physical function (WOMAC)							
Weight (kg)	–3.09 (–5.18, –0.99)	0.004	0.93 (–1.84, 3.71)	0.510	–4.02 (–5.77, –2.26)	<0.001	–

* Estimated mean adjusted for baseline mediator scores, baseline outcome scores and the stratifying variable, history of knee surgery (arthroscopy or contralateral arthroplasty). Negative effects favor the Diet + Exercise group. 95% CI = 95% confidence interval; NRS = numerical rating scale (rated 0–10, with higher scores indicating worse pain); WOMAC = Western Ontario and McMaster Universities Osteoarthritis Index (physical function subscale; rated 0–68, with higher scores indicating worse function).

† Potential mediator is change in (6 months minus baseline).

‡ Percent mediated not presented as the direct and indirect effects are in opposite directions (30).

Associations between changes in potential mediators and outcomes (Pathway C in Figure 1), where the change in the mediator may or may not be attributed to group allocation (i.e., intervention received), are shown in Supplementary Table 5, available on the *Arthritis Care & Research* website at http://onlinelibrary.wiley.com/doi/10.1002/acr.25140. A comparison of the results of the analyses for Pathways A and C in Figure 1 with the results from the causal mediation analyses are provided in Supplementary Table 6 (available at http://onlinelibrary.wiley.com/doi/10.1002/acr.25140). However, only the causal mediation analyses results were used to investigate mediation (33).

### Sensitivity analyses

Sensitivity analyses are presented in Supplementary Table 2. Supplementary Table 3 and Supplementary Figures 1–6 (available on the *Arthritis Care & Research* website at http://onlinelibrary.wiley.com/doi/10.1002/acr.25140) show the value of the sensitivity parameter (rho) required to change the direction of the indirect effect for each treatment comparison (mediator and outcome). Where the causal mediation analysis results suggested mediation may be present, the sensitivity analyses suggested the indirect effect changes to 0 and then reverses direction at values of rho of at least 0.2. Although these appear large, there is no cutoff value for rho to judge the robustness of the results to the violation of the ignorability assumption (32). However, omitting an observed confounder (the relevant mediator at baseline) reduced rho by at most 0.13, suggesting 0.2 is a large critical value so results appear robust to the violation of the ignorability assumption.

## DISCUSSION

This study aimed to evaluate mediators of the effects of 2 telehealth‐delivered exercise programs, including 1 program with and 1 program without a weight‐loss diet, on pain and physical function in people with knee OA. We found that reduced fear of movement and increased arthritis self‐efficacy may mediate the effects of exercise, and diet and exercise, on improvements in pain and physical function. In addition, weight loss may mediate the effects of diet and exercise on pain and physical function, compared to exercise only and to information only.

Our findings add to the limited similar research available. To our knowledge, only 3 previous studies have evaluated mediators of exercise for people with OA (11, 12, 34), and only 1 of those evaluated psychosocial potential mediators (34). This 1 study, by Rejeski and colleagues, found that self‐efficacy did not mediate the effects of exercise on physical function (34), which differs from our findings. However, Rejeski et al used a measure of self‐efficacy for stair climbing rather than for arthritis symptom management as was used in our study. In combined diet and exercise interventions compared to information alone, 2 previous studies found that self‐efficacy mediated effects on physical function (16) and that weight loss mediated effects on pain and physical function (13), both of which concur with our findings. We also found that weight loss may mediate the effects of our Diet + Exercise program on pain and function when compared to exercise only. To our knowledge, no previous studies have examined weight loss as a potential mediator of the effects of diet and exercise compared to exercise alone. Only 1 previous study (15) that examined mediators of a diet and exercise program compared to exercise only found that knee extensor strength and self‐efficacy for walking duration (neither of which were measured in our study) mediated effects on physical function and the 6‐minute walk test. Finally, we found that reduced fear of movement may mediate the effects of both exercise and diet and exercise on physical function. To our knowledge, no previous studies examined fear of movement as a potential mediator, so further research is required to confirm our findings.

We found no evidence of mediation by attitudes toward self‐management, physical activity, or willingness to have knee surgery, for either diet and exercise or exercise alone. Attitudes toward self‐management or willingness to have surgery have not previously been examined as potential mediators of exercise or diet and exercise programs. One previous study (35) examined physical activity as a potential mediator of a diet and exercise program for people with knee OA, also finding no evidence of mediation on changes in pain. This suggests that these variables may not contribute toward the mechanism of effect on symptoms of pain and function, or the tools used to measure these domains are problematic. Further research is needed to confirm our findings and to evaluate whether they have an important role in other outcomes such as quality of life, mental health, or health care costs. Future interventions could explore inclusion of components targeting “activation.”

Our findings have implications for the design of future exercise and diet programs for people with knee OA. We found that fear of movement and/or arthritis self‐efficacy have important roles in the mechanism of effect of exercise, and diet and exercise, programs. Intuitively, it makes sense that greater belief in one's capability to manage their OA, and reduced fear of engaging in exercising or physical activity, could contribute to greater improvements in self‐reported pain or physical function. Indeed, arthritis self‐efficacy has been found to have significant overall associations with pain severity and function (36, 37). There is also evidence that greater arthritis‐related self‐efficacy is associated with better overall health status and lower health care costs (38), suggesting that there are potentially additional benefits. Similarly, reduced fear of movement has been associated with lower pain and better physical function (39, 40, 41). Collectively, this suggests that future exercise, and diet and exercise, programs should include elements that target self‐efficacy and fear of movement. For example, self‐efficacy has been shown to be enhanced by various strategies like motivational interviewing (42, 43), pain‐coping skills training (44), education about self‐management (43, 45, 46), or a home‐based exercise program (45, 46), all of which were key components of both of our programs. In addition, education about OA using an empowerment and participatory discourse (rather than a disease and impairment discourse) has recently been shown to improve self‐efficacy and fear of movement and could be incorporated into the information provided in our interventions (47). Other behavioral and psychological interventions targeting fear of movement (48) may also be helpful additional components and contribute to further improved physical function.

We found that weight loss may mediate the effects of the diet and exercise program on outcomes when compared to control and to exercise alone. Our findings suggest that a 10 kg loss of weight (approximately proportional to a 10% reduction in weight, based on the mean weight of the cohort at baseline) corresponded to a 1.2‐unit (compared to control) and a 1.1‐unit (compared to exercise alone) improvement in pain (on the 0–10 NRS), and a 5.8‐unit and 4.8‐unit improvement in physical function (on the 0–68 WOMAC scale), respectively. These changes do not quite reach the minimal clinically important difference (MCID) for pain and function (1.8 units and 6.0 units, respectively [49]), though MCIDs vary depending on the characteristics of the population and the treatment they receive (49). Indeed, a recent systematic review that aimed to identify the most credible MCIDs for outcomes in those with chronic knee pain stated that their best estimates of MCIDs were ~10% of the instrument's total range (50), suggesting a change of 1 NRS unit in pain may be considered worthwhile. In addition, if combined with other mechanisms such as improvement in self‐efficacy and fear of movement, weight loss may contribute to clinically significant improvements in outcomes. Weight loss is thought to alter mechanical pathways and loading within the joint, contributing to reduced pain (5, 51). Weight loss has also been shown to lower joint inflammation and change levels of joint biomarkers (52, 53, 54), improving quality of life (including subdomains of physical functioning, vitality, stress, and mental health) (55). There are numerous diets which could facilitate weight loss in people with OA. Our study used a ketogenic VLCD, which has been perceived by participants to be easy to use, convenient, and effective (56). This is supported by other research suggesting that ketogenic VLCDs are associated with significant (10–16 kg) weight loss in people who are overweight or obese, which is maintained at 2 years follow‐up (57). Importantly, our diet program also involved a suite of information booklets and behavior change support that likely helped support weight loss (56), suggesting that future exercise and weight‐loss programs for this population may consider including similar components.

Our study has limitations. Our findings should be interpreted with caution, as other mediating factors, like joint loading, muscle strength, and inflammatory biomarkers (none of which were measured in our trial), may also account for part of the treatment effects (11, 14). As potential mediators were examined separately in this study, our analyses do not account for potential interactions between mediators. For example, it is not clear whether reduced fear of movement led to improved arthritis self‐efficacy, rather than the program itself being responsible for the change in self‐efficacy. In addition, there may be other confounders that were not accounted for; however, our sensitivity analyses suggested findings were insensitive to unmeasured mediator–outcome confounding. Our analysis was based on participants with complete data, and attrition rates differed across the 3 trial groups (6). This may have introduced bias if missing data were related to mediators or outcomes; however, the assumption that data were missing completely at random appeared reasonable since baseline characteristics were similar between those with missing data versus those with complete data. Our findings cannot necessarily be generalized to those without private health insurance or to those residing outside of Australia. Finally, our findings may also not be generalizable to exercise or diet programs that do not include the level of behavior change support included in our programs, such as use of motivational interviewing by clinicians, and provision of bespoke booklets with behavior change activities.

In conclusion, increased arthritis self‐efficacy, reduced fear of movement, and weight loss may partially mediate telehealth‐delivered exercise program effects, including those programs with and without diet, on pain and/or function in knee OA. Weight loss may partially mediate the effect of diet and exercise compared to exercise alone.

## AUTHOR CONTRIBUTIONS

All authors were involved in drafting the article or revising it critically for important intellectual content, and all authors approved the final version to be submitted for publication. Dr. Lawford had full access to all of the data in the study and takes responsibility for the integrity of the data and the accuracy of the data analysis.

### Study conception and design

Lawford, Hinman, Egerton, Keating, Brown, Oliver, Bennell.

### Acquisition of data

Lawford.

### Analysis and interpretation of data

Lawford, Hinman, McManus, Lamb, Bennell.

## Supporting information


Disclosure form

**Appendix S1:** Supplementary Information
