# Supplementary material for: How Does Exercise, With and Without Diet, Improve Pain and Function in Knee Osteoarthritis? A Secondary Analysis of a Randomized Controlled Trial Exploring Potential Mediators of Effects
Source: Arthritis Care Res (Hoboken). 2023 Jun 15;75(11):2316–27. doi: 10.1002/acr.25140 (PMC10952828; doi:10.1002/acr.25140)
Supplement: Supplementary file 2 — Appendix S1: Supplementary Information [file ACR-75-2316-s001.docx]

**Supplementary Table 1. Participant baseline characteristics by whether data was complete.**

|  | **Complete**  **(n = 345)** | **Incomplete/missing**  **(n = 69^)** |
| --- | --- | --- |
| Group |  |  |
| Control, n (%) | 45 (13.0%) | 22 (31.9%) |
| Exercise, n (%) | 137 (39.7%) | 35 (50.7%) |
| Diet+Exercise, n (%) | 163 (47.2%) | 12 (17.4%) |
| Age (years), mean (SD) | 65.0 (8.3) | 64.0 (8.1) |
| Female, n (%) | 193 (55.9%) | 34 (49.3%) |
| Body mass index (kg/m^2^), median (IQR) | 32.4 (30.1-35.3) | 34.6 (31.6-37.6) |
| Knee pain (NRS), median (IQR) | 6 (5-7) | 6 (5-6) |
| Physical function (WOMAC), mean (SD) | 23.0 (9.8) | 24.7 (10.0) |
| Attitudes towards self-management (PAM-13), median (IQR) | 44 (40-48) | 44 (40-47) |
| Fear of movement (BFMS), median (IQR) | 12 (10-14) | 13 (10-14) |
| Self-efficacy (ASES), mean (SD) | 20.7 (3.9) | 19.4 (3.7) |
| Weight (kg), mean (SD) | 95.1 (13.4) | 99.5 (15.3) |
| Physical activity (IPEQ), median (IQR) | 21.6 (12.0-33.5) | 16.6 (11.0-29.4) |
| Unwilling to have surgery*, n (%) | 99 (28.7%) | 16 (23.2%) |
| History of knee surgery (arthroscopy or contralateral arthroplasty), n (%) | 199 (57.7%) | 35 (50.7%) |

SD=standard deviation.

IQR=inter-quartile range (25^th^-75^th^ percentile).

NRS=numerical rating scale; rated 0-10, with higher scores indicating worse pain.

WOMAC=Western Ontario and McMaster Universities Osteoarthritis Index (physical function subscale); rated 0-68, with higher scores indicating worse function.

PAM-13=Patient Activation Measure; scored 13-52, with higher scores indicating greater patient activation.

BFMS= Brief Fear of Movement Scale for osteoarthritis; scored 6-24, with higher scores indicating greater fear.

ASES=Arthritis Self-efficacy Scale; scored 3-30, with higher scores indicating greater self-efficacy.

IPEQ=Incidental and Planned Exercise Questionnaire, version W; scored 0-128, with higher scores indicating higher levels of activity.

^1 participant requested withdrawal of all data from the study, hence, of 415 participants enrolled, data for 414 participants are presented here.

*Rated using a 5-point scale with terminal descriptors of ‘definitely not willing’ to ‘definitely willing’, with those indicating ‘probably not willing’ or ‘definitely not willing’ classified as unwilling to have knee surgery in the near future, and all other options classified as willing.

**Supplementary Table 2. Sensitivity analyses for indirect (mediation) effects.**

| Sensitivity analyses for mediation effects were conducted to investigate how robust the full causal mediation analyses results were to violations of the sequential ignorability assumption (i.e., that there is an unmeasured confounder related to both the mediator and the outcome). *The larger the sensitivity parameter, rho, needed to reverse the sign of the indirect effect, the less sensitive (and more robust) the causal mediation analyses results are to the violation of the sequential ignorability assumption [32]. This is because, given treatment was randomised, the sensitivity parameter, rho, represents the correlation between the error terms of the mediator and outcome models, a measure of the degree of unmeasured mediator-outcome confounding [30]). So, increasing values of rho* *at which the indirect effect is zero indicate that stronger unmeasured mediator-outcome confounding is required to reverse the sign of the indirect effect. As there is no cut-off value to determine how large rho must be to indicate insensitivity to unmeasured mediator-outcome confounding, a strong known confounder was removed from a subsequent sensitivity analysis [32]. The logic in removing a strong known confounder was to determine the maximum impact the absence of a strong known confounder had on the values of rho compared to when the confounder was accounted for. This difference was a maximum reduction of 0.13 in rho (i.e. a maximum reduction of 0.13 in the degree of unmeasured mediator-outcome confounding needed to change the indirect effect to zero and then reverse the direction of the indirect effect; Appendix 2 Table 2). So, absence of a strong known confounder reduced rho by at most 0.13. This difference, a maximum reduction of 0.13 in rho, was used to approximately but objectively assume that a strong unmeasured confounder, if present, may decrease rho by at most 0.13 [32]. Since 0.13 is less than the minimum value of rho, 0.2 (Appendix 2 Table 1), that our sensitivity analyses determined was required to change the indirect effect to zero and then the direction of the indirect effect (where our causal mediation analyses results suggested mediation may be present), our causal mediation analyses results appear insensitive to the assumed impact of a strong unmeasured mediator-outcome confounder and thus, robust to the violation of the sequential ignorability assumption.*  The results of these analyses are provided in Table 1 and Figures 1-6. Omitting an observed confounder (the relevant mediator at baseline) from the sensitivity analyses reduced rho by at most 0.13 compared to when the observed confounder was accounted for (Table 2). |
| --- |

**Supplementary Table 3. Sensitivity parameter, rho#, when the indirect effect is zero.**

|  | **Potential mediator^** | **Exercise vs Control** | | **Diet+Exercise vs Control** | | | **Diet+Exercise vs Exercise** | | |
| --- | --- | --- | --- | --- | --- | --- | --- | --- | --- |
| Knee pain (NRS) | | | | |  |  |  |  |  |
|  | Attitudes towards self-management (PAM-13) | -0.16 |  | -0.10 | |  | | ⸺ |  |
|  | Fear of movement (BFMS) | 0.15 |  | 0.09 | |  | | ⸺ |  |
|  | Self-efficacy (ASES) | -0.28 |  | -0.31 | |  | | ⸺ |  |
|  | Weight (kg) | 0.13 |  | 0.32 | |  | | 0.24 |  |
|  | Physical activity (IPEQ-W) | -0.10 |  | -0.09 | |  | | ⸺ |  |
|  | Unwilling to have surgery* | -0.10 |  | -0.20 | |  | | ⸺ |  |
| Physical function (WOMAC) | | | | |  |  |  |  |  |
|  | Attitudes towards self-management (PAM-13) | -0.08 |  | -0.18 | |  | | ⸺ |  |
|  | Fear of movement (BFMS) | 0.21 |  | 0.24 | |  | | ⸺ |  |
|  | Self-efficacy (ASES) | -0.28 |  | -0.48 | |  | | ⸺ |  |
|  | Weight (kg) | 0.09 |  | 0.36 | |  | | 0.26 |  |
|  | Physical activity (IPEQ-W) | -0.07 |  | -0.08 | |  | | ⸺ |  |
|  | Unwilling to have surgery* | 0.00 |  | -0.20 | |  | | ⸺ |  |

NRS=numerical rating scale; rated 0-10, with higher scores indicating worse pain.

PAM-13=Patient Activation Measure; scored 13-52, with higher scores indicating greater patient activation.

BFMS= Brief Fear of Movement Scale for osteoarthritis; scored 6-24, with higher scores indicating greater fear.

ASES=Arthritis Self-efficacy Scale; scored 3-30, with higher scores indicating greater self-efficacy.

IPEQ-W=Incidental and Planned Exercise Questionnaire, ‘past week’ version; scored 0-128, with higher scores indicating higher levels of activity.

WOMAC=Western Ontario and McMaster Universities Osteoarthritis Index (physical function subscale); rated 0-68, with higher scores indicating worse function.

#The larger the sensitivity parameter needed to reverse the sign of the indirect effect (as when the indirect effect is zero), the less sensitive (and more robust) the causal mediation analyses results are to the violation of ignorability assumption (which, given treatment was randomised, the sensitivity parameter, rho, represents the correlation between the error terms of the mediator and outcome models, a measure of the degree of unmeasured mediator-outcome confounding [30]).

^Potential mediator is change in (6 months minus baseline) except for the binary mediator, unwilling to have surgery, which is at 6 months.

*Rated using a 5-point scale with terminal descriptors of ‘definitely not willing’ to ‘definitely willing’, with those indicating ‘probably not willing’ or ‘definitely not willing’ classified as unwilling to have knee surgery in the near future, and all other options classified as willing.


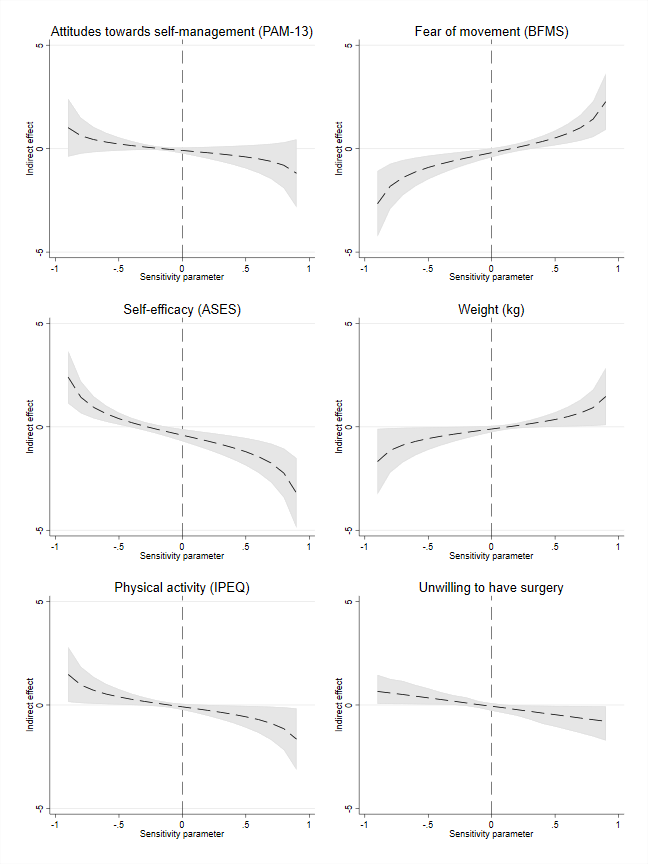


**Supplementary Figure 1.** The indirect (mediation) effect versus the sensitivity parameter (correlation between error terms of the mediator and outcome models) for each putative mediator of the Exercise program on change in knee pain (12 months minus baseline) compared to Control.

Note: the indirect effect through the putative mediator, self-efficacy, of Exercise compared to Control on change in knee pain is reversed below a sensitivity parameter of approximately -0.3.


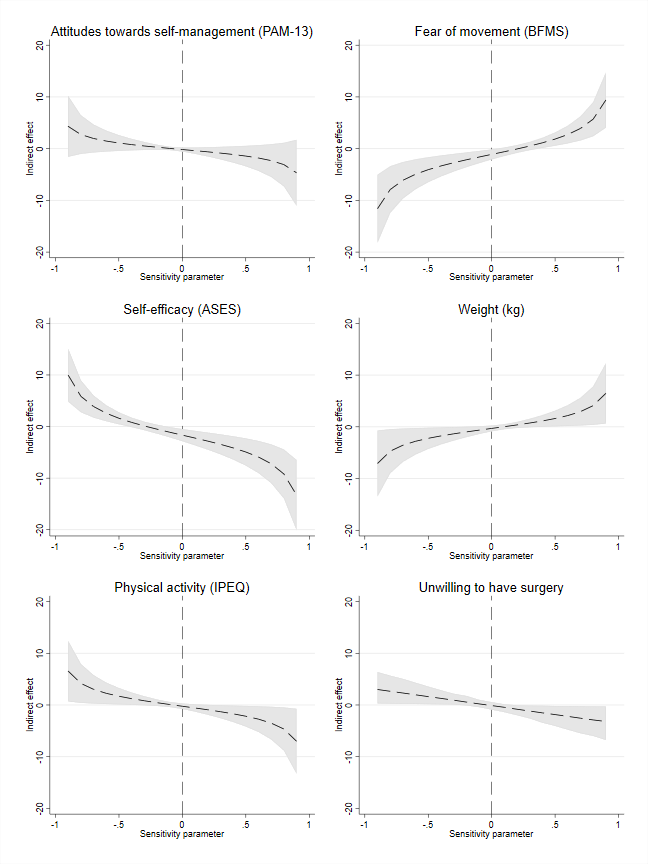


**Supplementary Figure 2.** The indirect (mediation) effect versus the sensitivity parameter (correlation between error terms of the mediator and outcome models) for each putative mediator of the Exercise program on change in physical function (12 months minus baseline) compared to Control.

Note: the indirect effect through the putative mediators, fear of movement and self-efficacy, of Exercise compared to Control on change in physical function is reversed above a sensitivity parameter of approximately 0.2 and below a sensitivity parameter of approximately -0.3 respectively.


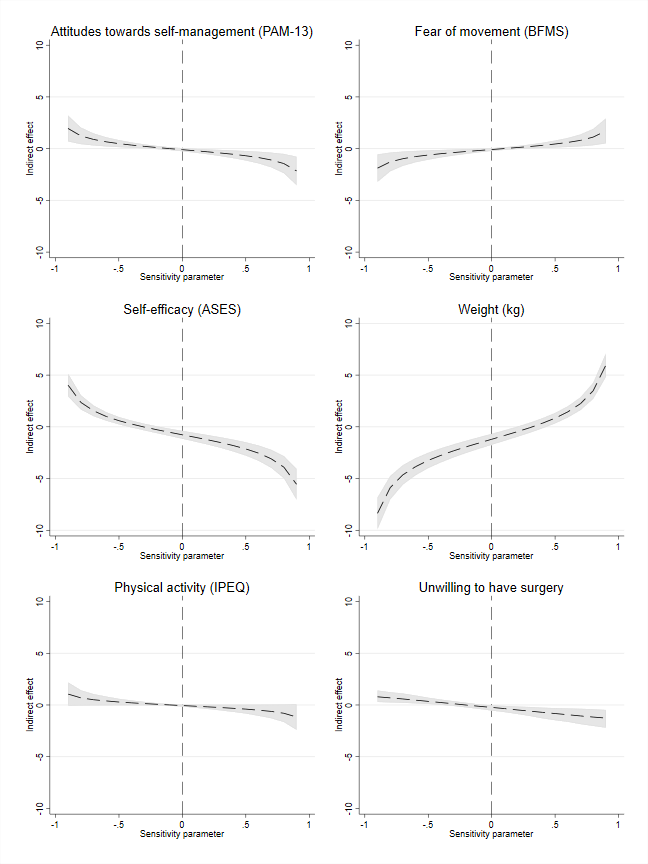


**Supplementary Figure 3.** The indirect (mediation) effect versus the sensitivity parameter (correlation between error terms of the mediator and outcome models) for each putative mediator of the Diet+Exercise program on change in knee pain (12 months minus baseline) compared to Control.

Note: the indirect effect through the putative mediators, self-efficacy and weight, of Diet+Exercise compared to Control on change in knee pain is reversed below a sensitivity parameter of approximately -0.4 and above a sensitivity parameter of approximately 0.3 respectively.

**Supplementary Figure 4.** The indirect (mediation) effect versus the sensitivity parameter (correlation between error terms of the mediator and outcome models) for each putative mediator of the Diet+Exercise program on change in physical function (12 months minus baseline) compared to Control.

Note: the indirect effect through the putative mediators, fear of movement, self-efficacy and weight, of Diet+Exercise compared to Control on change in physical function is reversed above a sensitivity parameter of approximately 0.3, below a sensitivity parameter of approximately -0.5 and above a sensitivity parameter of approximately 0.4 respectively.


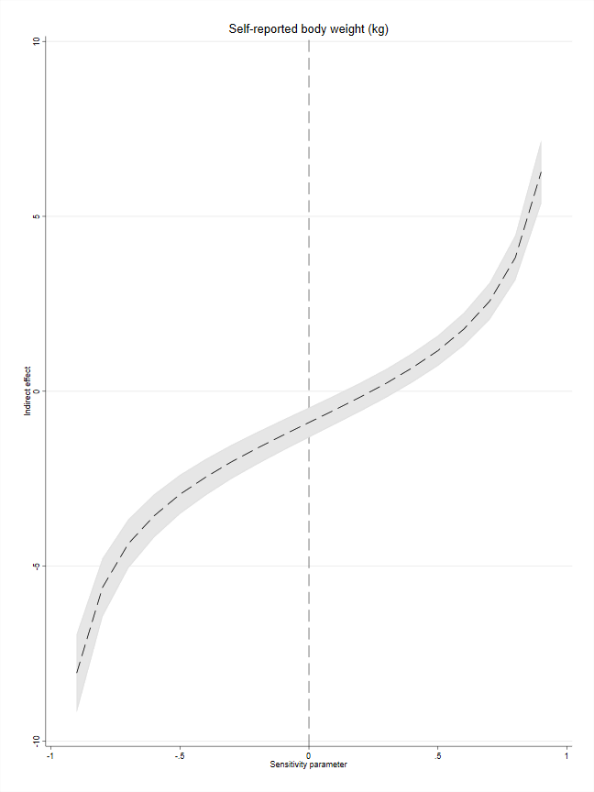


**Supplementary Figure 5.** The indirect (mediation) effect versus the sensitivity parameter (correlation between error terms of the mediator and outcome models) for the putative mediator, weight, of the Diet+Exercise program on change in knee pain (12 months minus baseline) compared to Exercise.

Note: the indirect effect through the putative mediator, weight, of Diet+Exercise compared to Exercise on change in knee pain is reversed above a sensitivity parameter of approximately 0.3.
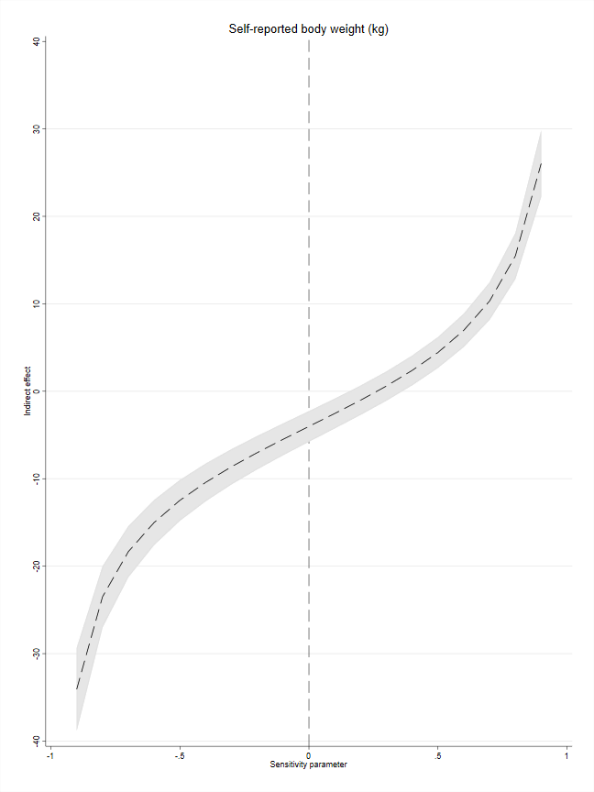


**Supplementary Figure 6.** The indirect (mediation) effect versus the sensitivity parameter (correlation between error terms of the mediator and outcome models) for the putative mediator, weight, of the Diet+Exercise program on change in physical function (12 months minus baseline) compared to Exercise.

Note: the indirect effect through the putative mediator, weight, of Diet+Exercise compared to Exercise on change in physical function is reversed above a sensitivity parameter of approximately 0.3.

**Supplementary Table 4. Sensitivity parameter, rho#, when the indirect effect is zero and an observed confounding variable (the relevant mediator at baseline) is omitted.**

|  | **Potential mediator^** | **Exercise vs Control** | | **Diet+Exercise vs Control** | | | **Diet+Exercise vs Exercise** | | |
| --- | --- | --- | --- | --- | --- | --- | --- | --- | --- |
| Knee pain (NRS) | | | | |  |  |  |  |  |
|  | Attitudes towards self-management (PAM-13) | -0.10 |  | -0.07 | |  | | ⸺ |  |
|  | Fear of movement (BFMS) | 0.04 |  | 0.03 | |  | | ⸺ |  |
|  | Self-efficacy (ASES) | -0.25 |  | -0.30 | |  | | ⸺ |  |
|  | Weight (kg) | 0.12 |  | 0.26 | |  | | 0.24 |  |
|  | Physical activity (IPEQ-W) | -0.11 |  | -0.07 | |  | | ⸺ |  |
|  | Unwilling to have surgery* | -0.10 |  | -0.20 | |  | | ⸺ |  |
| Physical function (WOMAC) | | | | |  |  |  |  |  |
|  | Attitudes towards self-management (PAM-13) | -0.02 |  | -0.12 | |  | | ⸺ |  |
|  | Fear of movement (BFMS) | 0.08~ |  | 0.14 | |  | | ⸺ |  |
|  | Self-efficacy (ASES) | -0.21 |  | -0.41 | |  | | ⸺ |  |
|  | Weight (kg) | 0.06 |  | 0.28 | |  | | 0.22 |  |
|  | Physical activity (IPEQ-W) | -0.06 |  | -0.04 | |  | | ⸺ |  |
|  | Unwilling to have surgery* | 0.00 |  | -0.20 | |  | | ⸺ |  |

NRS=numerical rating scale; rated 0-10, with higher scores indicating worse pain.

PAM-13=Patient Activation Measure; scored 13-52, with higher scores indicating greater patient activation.

BFMS= Brief Fear of Movement Scale for osteoarthritis; scored 6-24, with higher scores indicating greater fear.

ASES=Arthritis Self-efficacy Scale; scored 3-30, with higher scores indicating greater self-efficacy.

IPEQ-W=Incidental and Planned Exercise Questionnaire, ‘past week’ version; scored 0-128, with higher scores indicating higher levels of activity.

WOMAC=Western Ontario and McMaster Universities Osteoarthritis Index (physical function subscale); rated 0-68, with higher scores indicating worse function.

#The larger the sensitivity parameter needed to reverse the sign of the indirect effect (as when the indirect effect is zero), the less sensitive (and more robust) the causal mediation analyses results are to the violation of ignorability assumption (which, given treatment was randomised, the sensitivity parameter, rho, represents the correlation between the error terms of the mediator and outcome models, a measure of the degree of unmeasured mediator-outcome confounding [30]).

^Potential mediator is change in (6 months minus baseline) except for the binary mediator, unwilling to have surgery, which is at 6 months.

*Rated using a 5-point scale with terminal descriptors of ‘definitely not willing’ to ‘definitely willing’, with those indicating ‘probably not willing’ or ‘definitely not willing’ classified as unwilling to have knee surgery in the near future, and all other options classified as willing.

~The rho of 0.08 was 0.13 less than when the observed confounder, the mediator (fear of movement) at baseline, was included in the sensitivity analysis model.

**
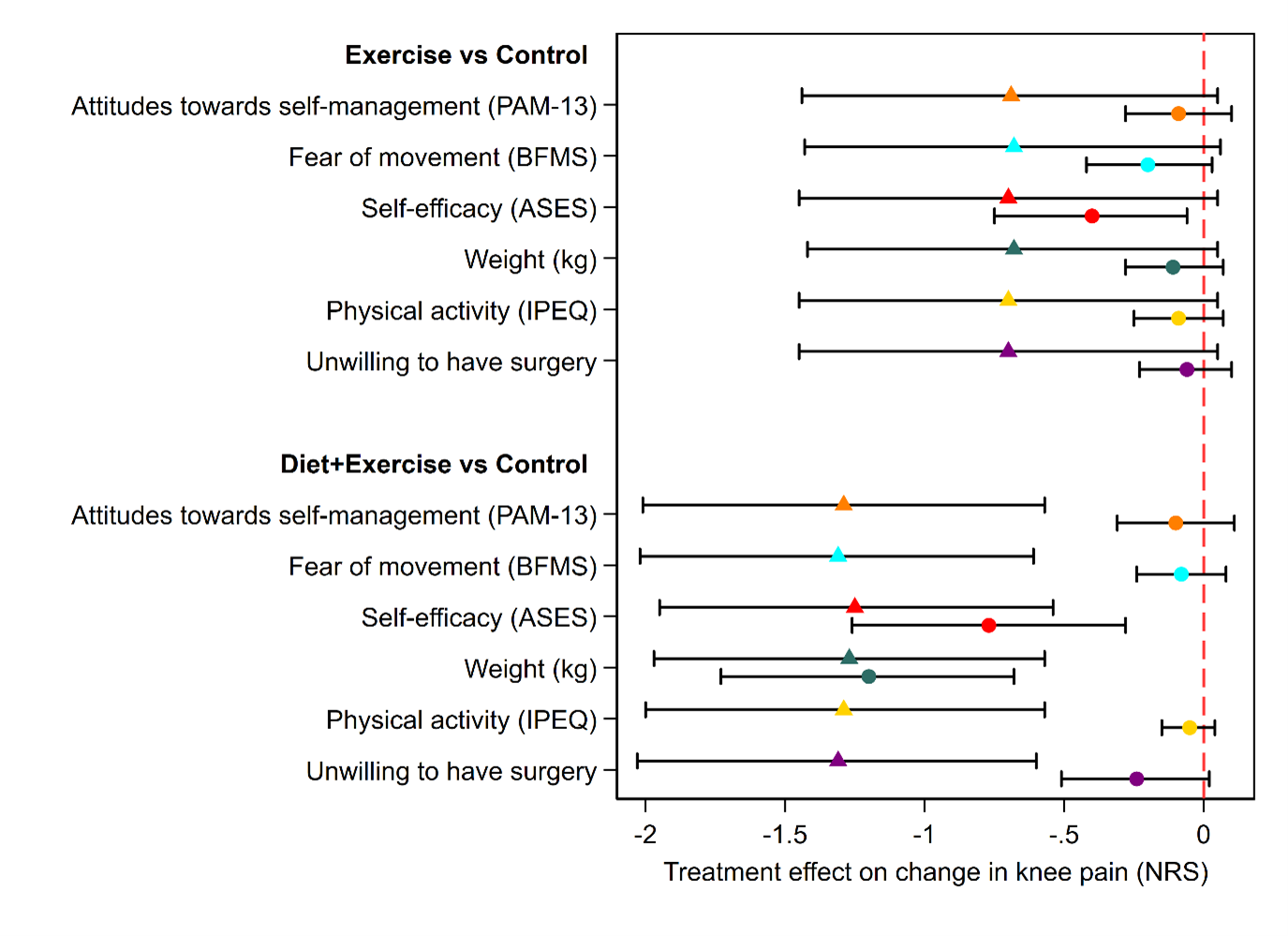
**

**Supplementary Figure 7. The total and indirect effects of *Exercise* and *Diet+Exercise*, compared to *Control*, on change (12 months minus baseline) in knee pain (NRS) mediated through the potential mediators (6 months minus baseline or at 6 months).** Triangles indicate total effects. Circles indicate indirect effects. Error bars indicate 95% confidence intervals.

**
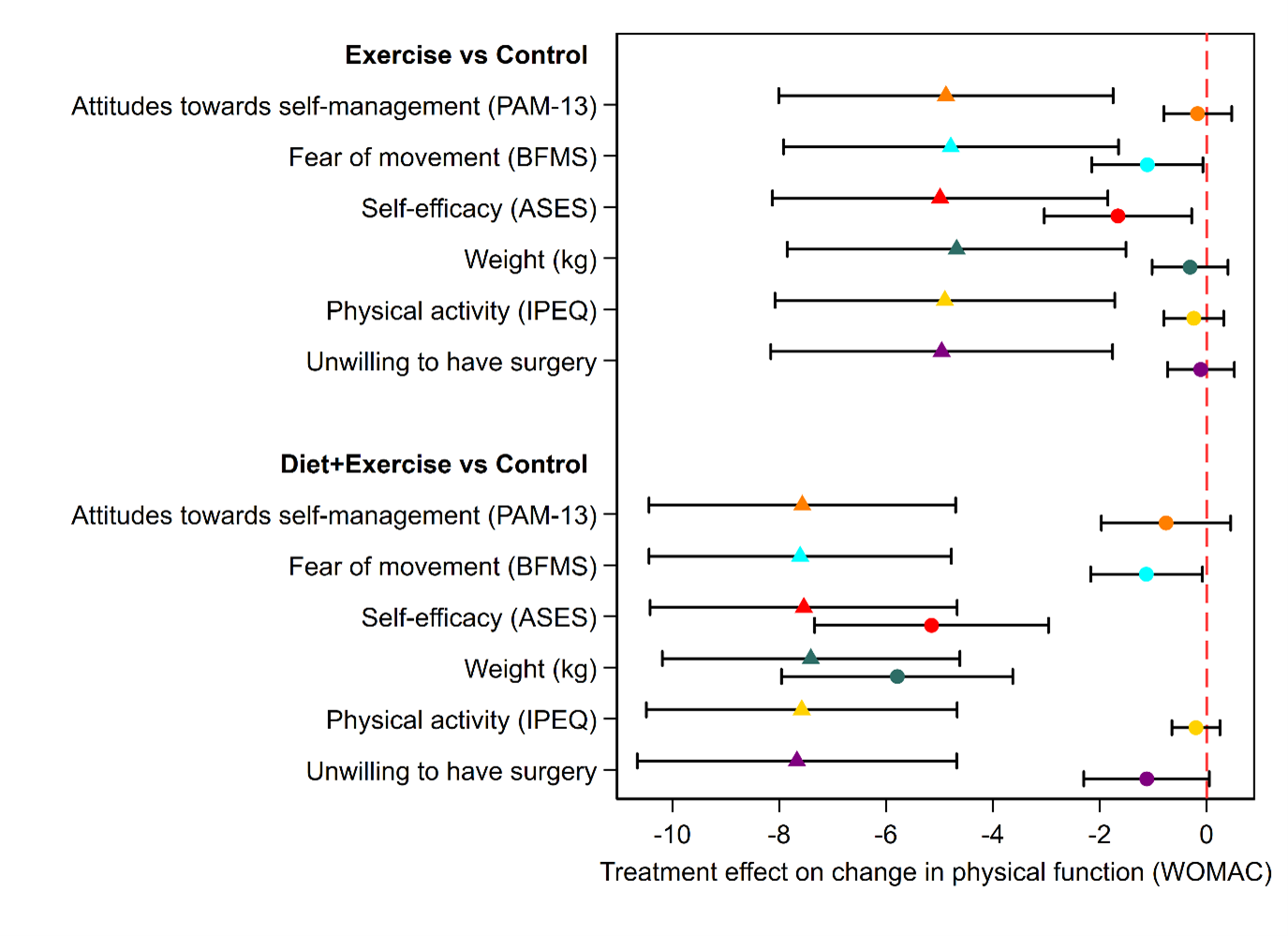
**

**Supplementary Figure 8. The total and indirect effects of *Exercise* and *Diet+Exercise*, compared to *Control*, on change (12 months minus baseline) in physical function (WOMAC) mediated through the potential mediators (6 months minus baseline or at 6 months).**

Triangles indicate total effects. Circles indicate indirect effects. Error bars indicate 95% confidence intervals.

# **Supplementary Table 5:** **Estimated mean (95% CI) effect* of the potential mediator on change in symptoms (12 months minus baseline).**

| **Outcome (Change in)** | **Potential mediator** | **Mean (95% CI)** | | | |  |  |
| --- | --- | --- | --- | --- | --- | --- | --- |
|  |  | ***Exercise* and *Control* arms only (n = 182)** | ***P*** | ***Diet+Exercise* and *Control* arms only (n = 208)** | ***P*** | ***Diet+Exercise* and *Exercise* arms only (n = 300)** | ***P*** |
| Knee pain (NRS) |  |  |  |  |  |  |  |
|  | Attitudes towards self-management (PAM-13)# | -0.07 (-0.13, -0.01) | 0.033 | -0.03 (-0.08, 0.01) | 0.168 | ⸺ | ⸺ |
|  | Fear of movement (BFMS)~ | -0.12 (-0.24, -0.01) | 0.038 | -0.05 (-0.14, 0.03) | 0.199 | ⸺ | ⸺ |
|  | Self-efficacy (ASES)# | -0.13 (-0.20, -0.07) | <0.001 | -0.16 (-0.23, -0.09) | <0.001 | ⸺ | ⸺ |
|  | Weight (kg)~ | -0.08 (-0.17, 0.01) | 0.080 | -0.12 (-0.17, -0.07) | <0.001 | -0.11 (-0.16, -0.06) | <0.001 |
|  | Physical activity (IPEQ-W)# | -0.02 (-0.04, 0.01) | 0.161 | -0.01 (-0.03, 0.01) | 0.203 | ⸺ | ⸺ |
|  | Unwilling to have surgery^ | -0.32 (-1.03, 0.40) | 0.389 | -0.73 (-1.36, -0.11) | 0.022 | ⸺ | ⸺ |
| Physical function (WOMAC) |  |  |  |  |  | ⸺ | ⸺ |
|  | Attitudes towards self-management (PAM-13)# | -0.13 (-0.37, 0.12) | 0.306 | -0.27 (-0.47, -0.06) | 0.010 | ⸺ | ⸺ |
|  | Fear of movement (BFMS)~ | -0.67 (-1.13, -0.21) | 0.004 | -0.63 (-0.97, -0.28) | <0.001 | ⸺ | ⸺ |
|  | Self-efficacy (ASES)# | -0.55 (-0.83, -0.27) | <0.001 | -1.08 (-1.35, -0.80) | <0.001 | ⸺ | ⸺ |
|  | Weight (kg)~ | -0.22 (-0.57, 0.13) | 0.218 | -0.58 (-0.79, -0.38) | <0.001 | -0.48 (-0.68, -0.28) | <0.001 |
|  | Physical activity (IPEQ-W)# | -0.04 (-0.12, 0.04) | 0.345 | -0.05 (-0.13, 0.04) | 0.272 | ⸺ | ⸺ |
|  | Unwilling to have surgery^ | -0.54 (-3.38, 2.29) | 0.708 | -3.29 (-5.96, -0.61) | 0.016 | ⸺ | ⸺ |

CI=confidence interval.

NRS=numerical rating scale; rated 0-10, with higher scores indicating worse pain.

PAM-13=Patient Activation Measure; scored 13-52, with higher scores indicating greater patient activation.

BFMS= Brief Fear of Movement Scale for osteoarthritis; scored 6-24, with higher scores indicating greater fear.

ASES=Arthritis Self-efficacy Scale; scored 3-30, with higher scores indicating greater self-efficacy.

IPEQ-W=Incidental and Planned Exercise Questionnaire, ‘past week’ version; scored 0-128, with higher scores indicating higher levels of activity.

WOMAC=Western Ontario and McMaster Universities Osteoarthritis Index (physical function subscale); rated 0-68, with higher scores indicating worse function.

*Adjusted for baseline mediator scores, baseline outcome scores, the stratifying variable, history of knee surgery (arthroscopy or contralateral arthroplasty) and each relevant pair of treatment groups.

#Mean (95% CI) effect of a 1-unit increase (an improvement) in the potential mediator (at 6 months from baseline) on change in symptoms (12 months minus baseline). Negative effects indicate improvement.

~Mean (95% CI) effect of a 1-unit decrease (an improvement) in the potential mediator (at 6 months from baseline) on change in symptoms (12 months minus baseline). Negative effects indicate improvement.

^Rated using a 5-point scale with terminal descriptors of ‘definitely not willing’ to ‘definitely willing’, with those indicating ‘probably not willing’ or ‘definitely not willing’ classified as unwilling to have knee surgery in the near future, and all other options classified as willing. Mean (95% CI) effect of unwillingness to have knee surgery at 6 months on change in symptoms (12 months minus baseline). Negative effects indicate improvement.

**Supplementary Table 6. Comparison of results for Pathways A and C with results from causal mediation analyses**

| Most results of the causal mediation analyses were consistent with the results from pathways A and C. For example, Diet+Exercise may affect weight compared to Exercise (pathway A, Table 2) and weight may affect pain and function (pathway C, Table 6). The causal mediation results were consistent with this as they showed that weight may mediate the effect of Diet+Exercise on pain and function compared to Exercise (Table 5). There were a few instances where the results of the causal mediation analyses were inconsistent with the results from pathways A and C: specifically, the causal mediation results do not suggest that fear of movement mediates the effect of Exercise on pain compared to Control (Table 3), whereas according to Table 2, Exercise may affect fear of movement compared to Control (pathway A) and fear of movement may affect pain (pathway C, Table 6); additionally, the causal mediation results do not suggest that unwillingness to have surgery mediates the effect of Diet+Exercise on pain or function compared to Control (Table 4), however according to Table 2, Diet+Exercise may affect unwillingness to have surgery compared to Control (pathway A) and unwillingness to have surgery may affect pain or function (pathway C, Table 6). It should be noted, however, that, as recommended, the results from pathway A and C were not considered when exploring whether mediation was present, only the results from the full causal mediation analyses were considered for this purpose [Reference: VanderWeele TJ. Mediation Analysis: A Practitioner's Guide. Annu Rev Public Health. 2016;37:17-32. doi:10.1146/annurev-publhealth-032315-021402] . |
| --- |
